# Network analysis to evaluate the impact of research funding on research community consolidation

**DOI:** 10.1371/journal.pone.0218273

**Published:** 2019-06-18

**Authors:** Daniel J. Hicks, David A. Coil, Carl G. Stahmer, Jonathan A. Eisen

**Affiliations:** University of California at Davis, Davis, California, United States of America; KU Leuven, BELGIUM

## Abstract

In 2004, the Alfred P. Sloan Foundation launched a new program focused on incubating a new field, “Microbiology of the Built Environment” (MoBE). By the end of 2017, the program had supported the publication of hundreds of scholarly works, but it was unclear to what extent it had stimulated the development of a new research community. We identified 307 works funded by the MoBE program, as well as a comparison set of 698 authors who published in the same journals during the same period of time but were not part of the Sloan Foundation-funded collaboration. Our analysis of collaboration networks for both groups of authors suggests that the Sloan Foundation’s program resulted in a more consolidated community of researchers, specifically in terms of number of components, diameter, density, and transitivity of the coauthor networks. In addition to highlighting the success of this particular program, our method could be applied to other fields to examine the impact of funding programs and other large-scale initiatives on the formation of research communities.

## Introduction

In 2004, the Alfred P. Sloan Foundation launched a program focusing on the “Microbiology of the Built Environment”, sometimes known as “MoBE”. The aims of this program were to catalyze research on microbes and microbial communities in human built environments, such as homes, vehicles, and water systems; and to develop the topic into a whole field of inquiry. Prior to 2004, many new developments (e.g., major advances in DNA sequencing technology) had catalyzed innovation in studies of microbes found in other environments (e.g., those living in and on humans and other animals, those found in the soil, those found in the oceans), but these innovations had not spread rapidly enough to studies of the microbes in the built environment. Similarly, many developments had occurred in studies of the built environment (e.g., the spread of low cost sensor systems), but focus had not yet been placed on the living, microbial components of built environments. This is not to say there had been no studies on the MoBE topic prior to 2004, but rather that the pace of advances in the area were modest at best compared to advances in other areas of microbiology and built environment studies. The MoBE area was founded on the belief that institutionally supported, integrated, trans-disciplanary scientific inquiry could address these shortfalls and lead to major benefits in areas such as indoor health, disease transmission, biodefense, forensics, and energy efficiency.

The Sloan Foundation’s program ultimately lasted 15 years and invested more than $50 million on work in the MoBE field. A key goal of this program was to bring together the highly disparate fields of microbiology (especially the area focused on studies of entire ecosystems of microbes) and building science (e.g. with a focus on building, maintaining, regulating, and studying built environments) with their different approaches, cultures, incentives, and rewards. Grants were given to many projects and a diverse collection of people covering many fields including microbiology, architecture, building science, software development, and meeting organization (a list of all grants from the program can be found at https://sloan.org/grants-database?setsubprogram=2). The products of these grants included a diverse collection of programs and projects, dozens of new collaborations, many novel and sometimes large data sets on various MoBE topics, new software and tools for MoBE studies, and hundreds of scholarly publications.

Recent reviews of the state of the field (e.g. [1] [2]) have qualitatively highlighted the success of this program. In this paper we report a quantitative assessment of the Sloan MoBE program and the MoBE field using a network analysis of scholarly literature. Specifically, the aim of this study was to compare the community of researchers funded by the Sloan Foundation’s MoBE program to their scientific peers. If the Sloan Foundation’s program was successful at cultivating a new research community around MoBE topics, we hypothesized that we would see the evolution of an increasingly dense and more tightly connected network over the duration of the funding program.

Programs explicitly dedicated to funding interdisciplinary research may have an important role to play in the development of new research communities. [3] finds that interdisciplinary research proposals are less likely to be funded by the Australian Research Council’s Discovery Programme, which is designed to fund basic research across the disciplines but is not explicitly interdisciplinary. This indicates an incentive for researchers to propose—and then conduct—disciplinary research, which is more likely to build on established research communities. By contrast, [4] finds evidence of both novel collaborations as well as cross-disciplinary citations and publications for researchers funded by the US National Robotics Initiative program, which is explicitly interdisciplinary.

[5] proposes that coauthor networks can be used to examine the emergence of Kuhnian “normal science” [6]. Specifically, they relate the formation of a giant component—in which a single connected component of the network contains a supermajority of authors—to the formation of the kind of research community Kuhn described. [5] focuses on three topological statistics for coauthor networks: (1) the diameter (average shortest path length between pairs of nodes) of the largest component, (2) the fraction of edges in the largest component, and (3) “densification,” the exponent of a power law model relating edge and node counts across time for a given dynamic network. While diameter and edge fraction are dynamic, calculated at each time step (e.g., annually) as the coauthor network changes, densification is a summary across time. [7] uses topic modeling to subdivide papers from the arXiv, the physics repository, into various subfields, then applies the approach of [5] to examine the dynamics of coauthor networks in each subfield. Following [5], [7] also uses the diameter of the largest component as a key statistic, but also examines the fraction of nodes, rather than edges, in the largest component.

As [5] acknowledges, Kuhn’s notion of a paradigm and normal scientific research is controversial. In addition, network topology alone cannot provide insight into the normative aspects of a Kuhnian paradigm. That is, in Kuhn’s view, a paradigm provides a rules and standards for good scientific research. The term paradigm comes from linguistics, in which a paradigm characterizes rules and standards for a specific construction. For example, “amo, amas, amat, amamus, amatis, amant” is a paradigm for the first conjugation of Latin verbs. Similarly, the paradigms for a normal science (e.g., protocols for experimental design and statistical analysis) provide shared rules and standards for good research—at least for the research community operating under the paradigm. The fact that a network of researchers are working with each other does not tell us whether they have this kind of shared normative framework.

However, the fact that a network of researchers are working with each other (or not) does provide insight into the structural possibilities for the circulation of ideas and information among researchers. Information flow within and across the boundaries of scientific communities has long been a major topic in science and technology studies (STS) and philosophy of science [8]; [9]; [10]. Increased information flow is also often a key goal of research funding programs, especially information flow across disciplinary boundaries [11]. Insofar as a scientific community is defined in terms of information flow, a transition from a disconnected or loosely-connected collaboration network to a highly-connected one does provide evidence for the formation of a scientific community.

[12] moves from coauthor networks to institutional collaboration networks (if X and Y are coauthors, then their respective institutions are collaborators) to examine the development of the field of strategic management. [12] calculates several dynamic network statistics for institutional networks, including average clustering, diameter, “connectedness” and “fragmentation” (which unfortunately are not defined, and have various incompatible definitions in the network analysis literature), and the number and fraction of nodes in the largest component.

[13] examines the role of funded researchers (“PIs”) in the collaboration network in Slovenia from 1970-2016. Part of their analysis focuses on the relationship among several statistics over overlapping time periods, including the fraction of nodes in the giant component, the mean fraction of each node’s neighbors who are PIs, the number of connected components when PIs are removed from the giant component, and the relative size of the largest component when PIs are removed.

All of these studies use dynamic analysis of coauthor networks to examine development and change in research communities over time. However, none of these studies is designed to examine the effect of a particular funding program on the research community, and only [13] situates the group of researchers of interest (“PIs” or funded researchers) in the context of their peers (i.e., authors who were not funded).

In contrast, [14] uses coauthor and institutional collaboration networks, among other bibliometric methods, to examine the impact of a US National Aeronautics and Space Administration (NASA) program focused on astrobiology; while [15] uses a coauthor network, again among other methods, to study the early impacts of the US National Science Foundation (NSF) Science of Science Policy (SciSIP) program. Because these are early assessments of their respective funding programs, both of these studies use static rather than dynamic collaboration networks.

[16] and [17] use dynamic network methods to analyze individual-level funding program impacts. [16] compares participants in two fellowship programs, funded by Japan Science and Technology Agency and Japan Society for the Promotion of Science, to their peers in a large literature database, focusing on individual betweeness centrality over time. [17] tests several hypotheses concerning the relationship between local topological features of the network (e.g., the size of a researcher’s neighborhood) and patent applications under a Chinese program to fund photovoltaic research.

Of these four program assessment studies, only [16] incorporates a comparison group of researchers.

In the present study, we use the theoretically-informed approach developed in [5] and [7] to examine the community-level impact of a specific funding program, namely, the MoBE program. By comparing MoBE-funded researchers to their peers, and incorporating robustness checks for the way peers are identified, we can have more confidence in the interpretation of our results as identifying causal effects of the MoBE program. In addition, by deploying a wider variety of network statistics, we identify changes in the coauthor networks that would be missed by the smaller set of statistics used in [5] and [7].

Compared to the literature reviewed above, our study is distinctive for using network analysis methods and a comparison group of researchers to analyze the community-level impacts of a particular research funding program. To be clear, we make no claims here about the impacts of research funding programs more generally, but we do think that the MoBE program is an interesting case of an explicit attempt to create an interdisciplinary, multi-institution research community. Insofar as we find that the MoBE program was successful in this attempt, future research might identify specific features of the program that contributed to this success and could be generalized to other such programs.

## Methods and materials

### Corpus selection

Publications funded by the Sloan Foundation’s MoBE program provided the starting point for our data collection and analysis. We evaluate the effect of this program by analyzing these publications in the context of previous work by the same authors, as well as a “control” or comparison set of authors working in the same general areas. We identify the comparison set as authors publishing frequently in the same journals as MoBE-funded publications.

### Identifying sloan foundation-funded publications

**Fig 1:**
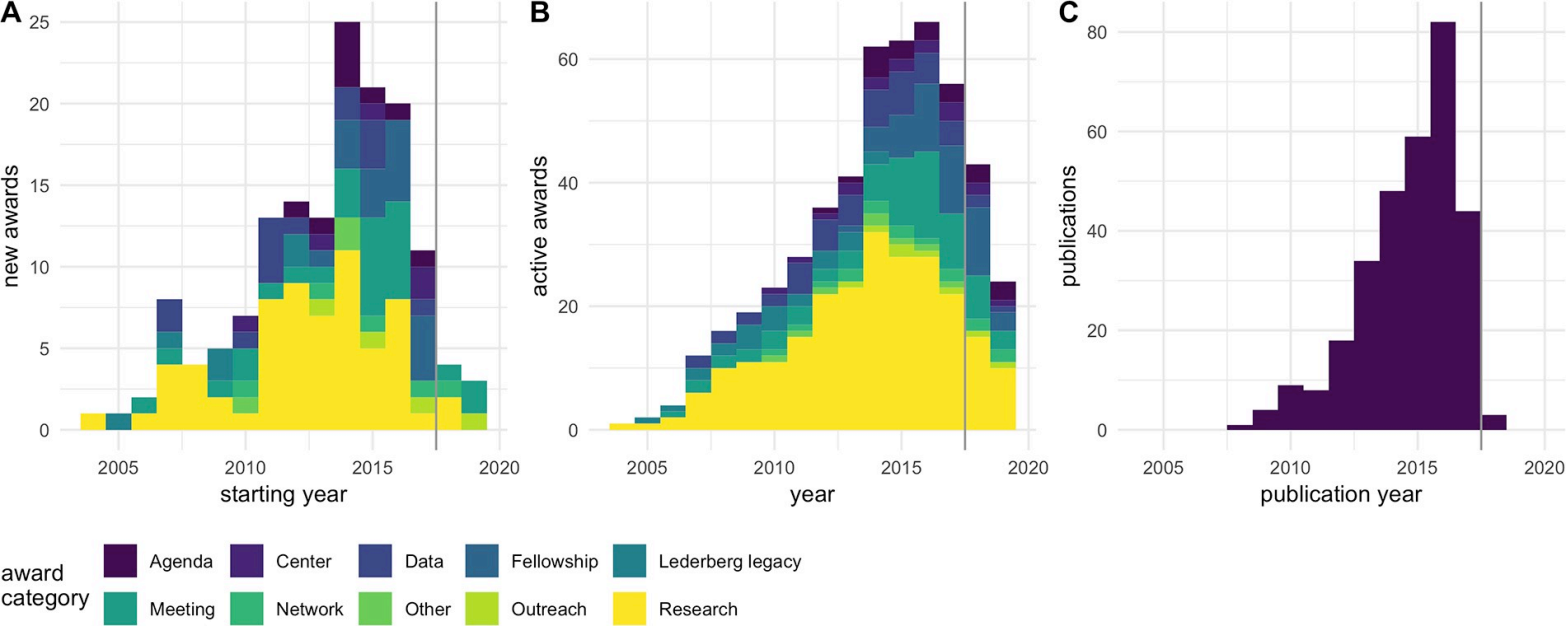
Awards and publications under the MoBE program. A: New awards made each year. B: Active awards in each year. C: Publications in each year. Dark gray vertical lines indicate the end of 2017, when MoBE-funded publications were identified. Colors indicate award types in A and B; color is not meaningful in C.

A list of awards made within the Sloan-funded MoBE program is available at https://sloan.org/grants-database?setsubprogram=2. The MoBE program awarded USD 51,000,000 in grants ranging from USD 3,500 to USD 2,500,000 (mean USD 335,000, median USD 125,000). Table 1 lists organizations than received 3 or more awards from this program. Fig 1 shows the number of new and active awards and publications within the MoBE program over time. While the earliest research awards were awarded in 2004, the number of new research awards expanded rapidly starting in 2011, with peak activity (most active research awards) in 2014. The first MoBE-funded publications did not appear until 2008, and peak publication occurred in 2016, indicating a lag of 2-3 years between research activities and the publication record.

**Table 1:** Organizations that received 3 or more awards under the MoBE program. Awards include research funding as well as funds for meeting organization, data infrastructure development, outreach, and other categories. n: Number of awards received.

organization	n
University of Colorado, Boulder	15
University of California, Berkeley	12
The University of Chicago	7
University of California, Davis	7
University of Oregon	7
Yale University	7
The University of Texas, Austin	5
Virginia Polytechnic Institute and State University	5
J. Craig Venter Institute	4
Marine Biological Laboratory	4
National Academy of Sciences	4
Cornell University	3
Harvard University	3
Illinois Institute of Technology	3
Ohio State University	3
University of California, San Diego	3
University of Maryland, Baltimore	3
University of Toronto	3

A list of publications associated with the MoBE program was compiled through a combination of strategies. An initial set of papers was identified by manually searching for acknowledgement of Sloan Foundation funding in any publications authored by the grantees during the program period. Additional publications were identified by searching Google Scholar for relevant MoBE papers and identifying those authored by grantees during the program period. Finally, each grantee (as well as sometimes their lab members (n = ^~^50)) was contacted directly and asked whether the publication list we had for them was both accurate and complete. This feedback led to some publications being removed from the list (as having not derived from the Sloan Foundation’s program) and others being added. In addition, we posted requests for feedback in various social media settings (e.g., blogs, Twitter) asking for feedback on the list (https://www.microbe.net/2017/09/07/sloan-funded-mobe-reference-collection/; https://www.microbe.net/2018/03/15/one-last-call-for-help-with-sloan-funded-mobe-paper-collection/). The final list contained 327 publications. 20 of these publications did not have digital object identifiers (DOIs) on record and were excluded from further analysis.

### Identifying peer authors

We sought to compare MoBE researchers to peers who were not funded by the MoBE program, in order to control for ordinary developments in both individual careers (e.g., more senior researchers are likely to have more collaborators) and research communities (e.g., more researchers are trained and join the community). In what follows, researchers funded by the Sloan Foundation’s program are referred to as the “collaboration” authors; their peers are the “comparison” authors.

Several methods were considered for developing this comparison set. Keyword searches were judged to be too noisy, producing significant numbers of false positive and false negative matches, as well as highly sensitive to the particular keywords used. Forward-and-backward citation searches using the 307 MoBE articles (compare [18]) produced lists on the order of 1,000,000 publications, which was judged to be impractically large. As an alternative, peer authors were identified as authors who are highly prolific in the same journals as the 307 MoBE articles.

**Fig 2:**
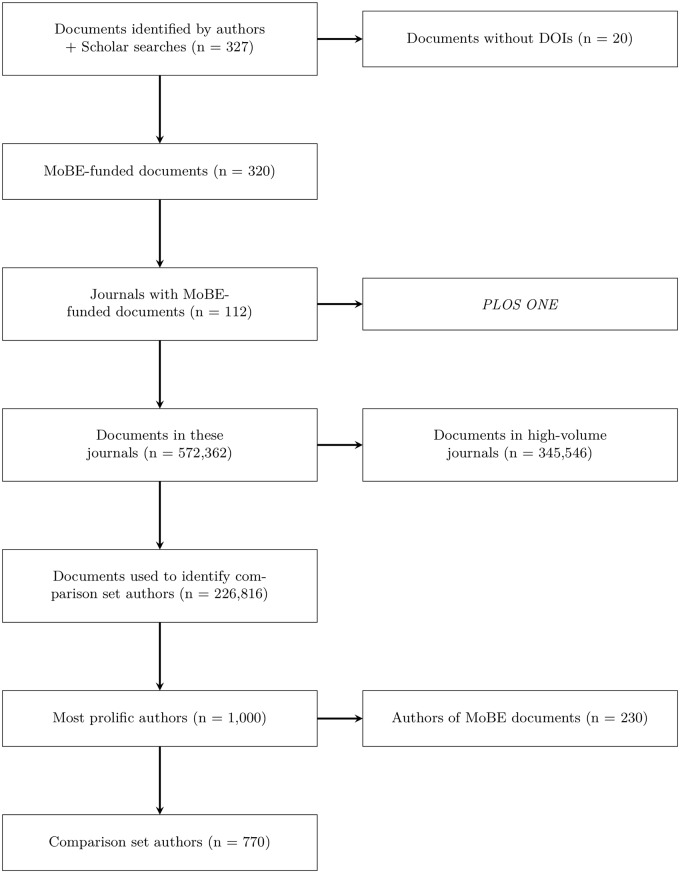
Flow diagram for comparison set construction.

Specifically, using the rcrossref package [19] to access the Crossref API (application programming interface; https://github.com/CrossRef/rest-api-doc), metadata were retrieved for 572,362 articles published in 111 journals between 2008 and 2018 inclusive. (*PLOS One* was dropped prior retrieving these metadata, due to its general nature and extremely high publication volume.) 14 journals published at least 10,000 articles during this time period; these appeared to be high-volume, general or broad-scope journals, such as *Science* or *Environmental Science & Technology*. The 345,546 articles from these 14 journals were removed, leaving 226,816 articles from 97 journals. Because Crossref does not provide any standardized author identifiers, simple name matching was used to estimate the number of articles published by each author. (This method means “Maria Rodriguez” and “M. Rodriguez” would be counted as different authors at this stage.) The same method was used to roughly identify authors of MoBE-funded papers. After filtering out authors of MoBE-funded papers, the 1,000 most prolific authors were selected as candidates for the comparison set. See Fig 2.

Next, to retrieve standardized author identifiers, a covering set of papers was identified such that each candidate name appeared as an author of at least one paper in the covering set. This covering set included all candidates by name, and no filtering was applied in identifying the covering set. Metadata for these papers was retrieved from the Scopus API (https://dev.elsevier.com), which incorporates an automated author matching system and standardized identifiers, referred to as author IDs. These author IDs were then used to characterize researchers as members of the MoBE collaboration or comparison set. Collaboration authors were defined as any author who either (a) was an author of at least two MoBE-funded papers or (b) was the author of at least one MoBE-funded paper and appeared in the candidates list (total n = 393 distinct names for the collaboration; 438 distinct author IDs). Candidates for the comparison set were removed if they were classified as part of the collaboration (total n = 770 distinct author IDs for the comparison set). (In what follows, we do not distinguish between authors and author IDs).

### Author histories

Author histories (up to 200 publications since 1999 inclusive) for all 1,208 authors were retrieved using the Scopus API. These histories include both MoBE-funded and non-MoBE-funded papers, published in all journals indexed by Scopus. This resulted in an analysis dataset of 85,306 papers. Besides standard metadata, each paper was identified as MoBE-funded (or not). Table 2 shows the distribution of papers in the analysis dataset across 4 author combinations: only comparison authors; only collaboration authors, with separate counts for MoBE and non-MoBE funded papers; and “mixed” papers, with authors from both sets.

**Table 2:** Counts of papers in the analysis dataset, grouped by author type and whether they were funded by the MoBE program. Author groups are based only on authors included in either the collaboration or comparison set. For example, a non-MoBE paper by two collaboration authors and a third author (not included in either the collaboration set or the comparison set) would be counted as “collaboration authors only”.

Paper group	n
Comparison authors only	67030
Collaboration authors only, non-MoBE	14610
Mixed comparison-collaboration, non-MoBE	1938
MoBE funded	286

### Disciplinary identification

As discussed in the introduction, one of the primary aims of the MoBE program was to promote interdisciplinary collaboration between microbiologists, on the one hand, and researchers in fields such as civil engineering and indoor air quality, on the other. To assess the success of the program in this respect, we attempted to collect data on researchers’ disciplinary self-identification. We contacted 80 MoBE-funded researchers via email, asking them what percentage of their research/work they would consider related to microbiology, building science, or “other.” 30 researchers responded. We conducted an exploratory analysis, looking for associations between area self-identification and researchers’ publications in the analysis dataset, based on (a) the All Science Journal Classification [ASJC] subject areas identified by Scopus, (b) all words used in paper abstracts, and (c) the 1000 most-informative words used in paper abstracts (where “informative” was calculated in terms of entropy over the self-identified disciplines). In each case, principal component analysis indicated that there were no useful associations that could be used to classify all authors within this disciplinary space (e.g., using a machine learning model). In light of these unpromising exploratory results and limited resources, efforts to interdisciplinary collaboration were not pursued further.

### Network analysis

The analysis dataset of 85,306 papers was used as the basis for constructing time-indexed collaboration networks. Each author forms a node (distinguished by author ID); edges correspond to papers published in a given year, so that two authors are connected by an edge for a given year if they coauthored at least one paper published in that year. All collaboration authors had at least one edge; 72 comparison authors did not have at least one edge (i.e., at least one paper coauthored with another author in the dataset), and were dropped from the network analysis (remaining comparison n = 698). Authors who collaborated on multiple papers in a given year were connected with multiple edges, except when calculating density (see below).

After constructing the combined (collaboration + comparison) network, separate cumulative-annual networks were constructed for each set of authors. For example, two authors would be connected in the 2011 network if and only if (1) they were in the same author set and (2) they had coauthored at least one publication between 1999 and 2011 inclusive. Cumulative networks were used to reduce noise in the most recent years, due to incomplete data for 2018 and as the Sloan Foundation’s funding program was starting to wind down. Analyzing separate cumulative networks allows the examination of the development of research communities through time and between the author sets.

For network analysis, we extended the approach developed by [5] and [7]. Specifically, both of these studies proposed that community formation can be measured in terms of giant component coverage and mean distance or shortest path length: increasing coverage combined with decreased distance indicates community consolidation. Neither [5] nor [7] used a control or comparison group (neither study aimed to to examine the impact of a specific funding program or other intervention). In the study, we calculated a total of eight network topological statistics and directly compare the two author sets. Specifically, we calculated the number of authors, number of components, coverage of the giant component (as a fraction of authors included in the largest component), entropy (*H*) of the component size distribution, diameter, density (fraction of all possible edges actually realized), mean distance, and transitivity in each year.

Number of authors simply measures the total size of each network. Because these are cumulative networks, the number of authors necessarily increases. The number of components, coverage of the giant component, and entropy of the component distribution are measures of the large-scale structure of the network. More components indicate that the network is divided into subcommunities that do not interact (at least in terms of coauthoring papers); fewer components indicates consolidation of the research community. Giant component coverage and entropy measure the relative sizes of these different components; higher giant component coverage and lower entropy indicate that more authors can be found in a single component, which in turn indicates research community consolidation.

Diameter, density, and mean distance can be interpreted as measures of the ability of information to flow through the network. Lower diameter, higher density, and lower mean distance indicate that it is easier for information to move between any two given researchers, as there are fewer intermediary coauthors and a higher probability of a direct connection. These therefore indicate research community consolidation.

Transitivity is an aggregate measure of the local-scale structure of the network. Low transitivity indicates that the network is comprised of loosely connected clusters; there is collaboration across groups of researchers, but it is relatively rare. High transitivity, by contrast, indicates that the network cannot be divided into distinguishable clusters. High transitivity therefore indicates research community consolidation.

Two robustness checks were incorporated into our analysis. First, to account for the possibility of data errors or missingness, perturbed networks were generated for each year by randomly switching the endpoints of 5% of edges. Second, the construction of the comparison set is likely to exclude students, postdoctoral researchers, and other early-career researchers. Insofar as these types of authors are included in the collaboration set, the collaboration network may appear to be more well-connected than the comparison set. To account for this possibility, we construct and analyze filtered versions of the annual cumulative networks. Authors are included in the filtered versions only if they have 50 or more papers total in the analysis analysis dataset.

Acknowledgment sections and other sources of funding information are not included in the metadata retrieved for this analysis. We are therefore unable to identify funding sources except for MoBE-funded papers, for which we have our own metadata. The comparison method is thus designed to test only whether or not the removal of MoBE-funded research produces a response effect in the shape of the overall discursive space. It does not consider independent relationships between MoBE and other sources nor relationships between non-MoBE sources. An underlying assumption of the analysis is, therefore, that the rates of impact from other sources of research funding are constant and that there is no underlying relationship between MoBE funding and other funding sources such that the removal of MoBE funding results in uneven removal of another source(s) of funding. Examining these relationships is potential direction for future study.

All data collection and analysis was carried out in R [20]. Complete data collection and analysis code, as well as the list of MoBE-funded publications, is available at https://doi.org/10.5281/zenodo.2548839.

## Results/Discussion

### Qualitative analysis

**Fig 3:**
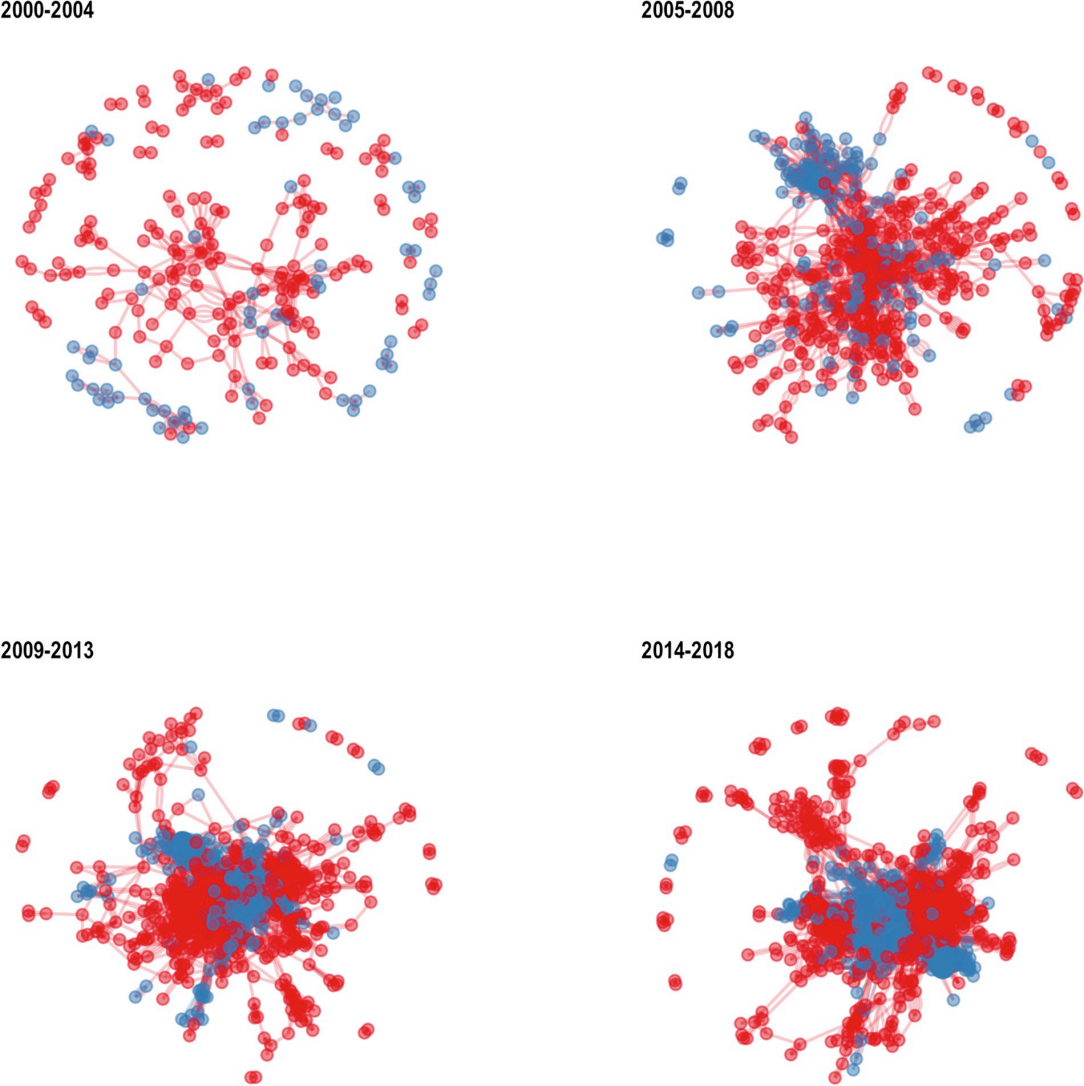
Consolidation of the MoBE collaboration over time. Panels show time slices (non-cumulative) of the giant component of the combined coauthor network. Blue nodes and edges are MoBE authors and papers; red nodes and edges are non-MoBE authors and papers. Network layouts are calculated separate for each slice using the Fruchterman-Reingold algorithm with default values in the igraph package.

The development of the combined network is shown in Fig 3. MoBE-funded authors and papers are shown in blue; non-MoBE-funded authors and papers are shown in red. All together, we believe that Fig 3 shows the consolidation of the MoBE collaboration within a consolidating larger research community.

Prior to the beginning of the MoBE funding in 2004, subset of MoBE researchers are actively working with each other; but many MoBE researchers are isolated in this network, and the largest component is only loosely connected. Qualitatively, the combined network has a sparse “lace” structure, with many long loops, as well as an “archipelago” of numerous small disconnected components.

During the early years of the funding period (2005-2008 and 2009-2013), a tighter cluster of MoBE researchers appears on the margins of the overall research community; but many MoBE researchers can be found scattered among the comparison authors and in disconnected components. The combined network has a “hairy ball” appearance, with a dense central “ball” and many peripheral “hairs,” and again an extensive “archipelago.” Part of the MoBE collaboration appears as a somewhat coherent “sub-ball.” We infer that this indicates that this part of the MoBE collaboration is highly integrated within the larger community.

During the peak period of MoBE funding (2015-2018), the vast majority of MoBE researchers appear to form one or two large, coherent communities at the center of the giant component—well-defined “blobs” of blue within a larger blob of red. Very few MoBE researchers appear outside of this coherent community. We suggest that this indicates tight integration involving almost all members of the MoBE collaboration.

However, because qualitative features of a visualized network are heavily dependent on the visualization method, this qualitative analysis should not be overinterpreted. Below we provide a quantitative analysis, less susceptible to overinterpretation.

Note that a few comparison set authors remain in small disconnected components even in the final time slice. These likely reflect “false positives” in the construction of the comparison set: authors who appear relatively frequently in the same journals as the MoBE publications, but do not actually conduct research in relevant research areas. We manually identified some such false positives, including authors of news stories in journals such as *Current Biology* or *Nature Biotechnology* as well as a few neuroscientists.

### Quantitative analysis

**Fig 4:**
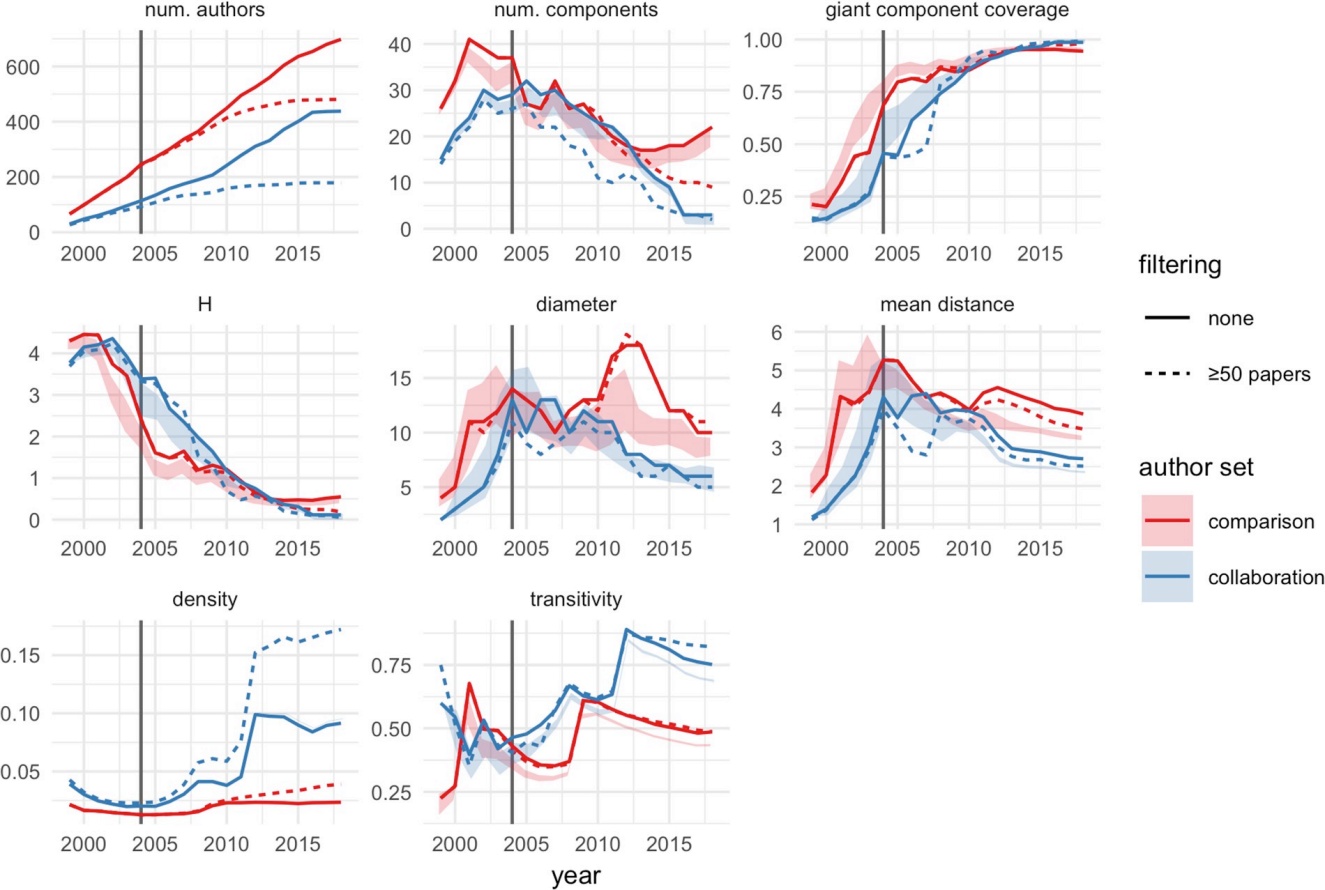
Network statistics over time. See text for explanation of the different statistics calculated here. Solid lines correspond to observed values; shaded ribbons correspond to 90% confidence intervals on rewired networks, where 5% of the observed edges are randomly rewired while maintaining each node’s degree distributions. 100 rewired networks are generated for each author set-year combination. Dashed lines correspond to observed values for authors with 50 or more total papers in the data. Blue corresponds to the MoBE collaboration; red corresponds to the peer comparison set of authors. Vertical lines indicate 2004, the first year of research funding by the MoBE program. Due to publication lags, we would not expect to see effects from 2004 funding until 2006-07.

Fig 4 shows statistics over time for the cumulative collaboration networks in each author set. Overall, both the MoBE research community and the comparison research community consolidated over time; but the MoBE research community consolidated faster and more thoroughly than the comparison set.

The most notable differences between the two author sets appear with the number of components, diameter, density, and transitivity. The comparison set stabilizes at 15-20 distinct components, while the MoBE collaboration approaches fewer than 5 components. However, for both author sets giant component coverage approaches 1 and *H* approaches 0, indicating that both networks contain a single giant component; the comparison set simply has several disconnected components with isolated researchers. As observed in the qualitative analysis, we believe this is plausibly due to “false negatives” in constructing the comparison set. The remaining statistics are generally robust to the inclusion of such “false negatives”.

Prior to 2010, the MoBE and comparison sets have a similar diameter: increasing during 1999-2005 as new researchers are added; then roughly stable until about 2010. Diameter remains above 10 for the comparison set, with a notable increase in 2008 followed by a decrease after 2013. By contrast, starting around 2010, the MoBE collaboration diameter is consistently less and decreasing.

However, diameter might be criticized as sensitive to network size. The relatively low diameter of the MoBE collaboration might be explained by the fact that this network has about half as many researchers as the comparison set.

Density and transitivity are automatically normalized against network size, and so avoid this potential confounder. For the collaboration set, transitivity peaks near 90% in 2012, indicating that at this time the connected components of the MoBE collaboration have almost no internal structure: everyone involved in the collaboration in 2012 is working directly with almost everyone else. Density plateaus at about 10% at this same time, and remains roughly stable over the remaining years of the study period. Transitivity and density then drop somewhat, but still remain remarkably high, indicating a highly interconnected research community even as the number of authors approaches its peak of just over 400. Transitivity is greater than 60% for both author sets in 2008-2009, but then diverges, dropping to around 50% in the comparison set by 2018. Density is consistently below about 2.5% for the comparison set throughout the entire study period.

Because of the delay between research and journal article publication, these network statistics provide a lagging indicator of community formation, of roughly 2-3 years. Taking this lag into account, our network analysis indicates that the MoBE research community consolidated around the period 2008-2010.

Shaded regions in Fig 4 indicate that most comparisons between the MoBE and comparison sets are robust to data errors. Diameter and number of components are somewhat more sensitive to possible data errors than the other statistics; but even here the comparison set statistics are consistently greater than the MoBE set statistics, indicating less consolidation in the comparison set.

The dashed lines in Fig 4 indicate that the comparisons are also robust to excluding early-career researchers. Other than the number of authors—which necessarily will decrease when authors are filtered—the only noteworthy effect of filtering is to increase the density of the collaboration network. There is no practical difference in the other statistics, especially for comparing the two networks of authors. Intuitively, filtering less productive authors is likely to remove less-connected authors from the margins of the network. These authors are less likely to provide important ties connecting otherwise separated communities.

## Conclusions

Overall, we believe our results support the hypothesis that the Sloan Foundation-funded researchers consolidated as a community over the course of the program during 2008-2010. Whereas at the start of the program there were relatively few connections between researchers, especially across domains, by the end of our study period the network was dense and highly interconnected. In particular, while the Sloan Foundation-funded community was initially less connected than the control community it reached a similar level of consolidation by the end of the study period. This suggests to us that the program was successful in the stated goal of increasing collaboration between researchers.

We note that the most dramatic differences between the MoBE collaboration and the comparison set could not have been detected using the two statistics calculated by [7], namely, giant component coverage and mean distance. Giant component coverage approached unity for both networks, and the difference in mean distance was relatively small. Mean distance could also be criticized as too sensitive to network size. By contrast, the most striking differences in this case appeared in density and transitivity, which are automatically normalized for network size.
